# A homozygous variant in cardiac troponin I3, TNNI3, causes severe pediatric restrictive cardiomyopathy

**DOI:** 10.1016/j.xhgg.2026.100598

**Published:** 2026-03-30

**Authors:** Jirko Kühnisch, Cara L. Barnett, Josephine Brendel, Lara Berklite, Chet Villa, Wenke Seifert, Sabine Klaassen, Karin Klingel, K. Nicole Weaver

**Affiliations:** 1 Experimental and Clinical Research Center, A Cooperation Between the Max Delbrück Center for Molecular Medicine in the Helmholtz Association and Charité - Universitätsmedizin Berlin, Germany; 2 Institute of Physiology, Brandenburg Medical School (MHB) Theodor Fontane, Brandenburg an der Havel, Germany; 3 Heart Institute, Cincinnati Children’s Hospital Medical Center, Cincinnati, OH, USA; 4 Charité - Universitätsmedizin Berlin, Corporate Member of Freie Universität Berlin und Humboldt-Universität zu Berlin, Berlin, Germany; 5 Division of Pathology, Cincinnati Children’s Hospital Medical Center, Cincinnati, OH, USA; 6 Department of Pathology and Laboratory Medicine, University of Cincinnati College of Medicine, Cincinnati, OH, USA; 7 The Heart Institute, Cincinnati Children’s Hospital Medical Center, Cincinnati, OH, USA; 8 Institute of Cell Biology and Neurobiology, Charité - Universitätsmedizin Berlin, Corporate Member of Freie Universität Berlin und Humboldt-Universität zu Berlin, Berlin, Germany; 9 DZHK (German Centre for Cardiovascular Research), Partner Site Berlin, Berlin, Germany; 10 Department of Congenital Heart Disease, Deutsches Herzzentrum der Charité (DHZC), Berlin, Germany; 11 Cardiopathology, Institute for Pathology and Neuropathology, University Hospital Tübingen, Tübingen, Germany; 12 Division of Human Genetics, Cincinnati Children’s Hospital Medical Center, Cincinnati, OH, USA; 13 Department of Pediatrics, University of Cincinnati College of Medicine, Cincinnati, OH, USA

**Keywords:** TNNI3, troponin, restrictive cardiomyopathy, dilated cardiomyopathy

## Abstract

Dilated cardiomyopathy (DCM) results from systolic dysfunction, while restrictive cardiomyopathy (RCM) is due to diastolic dysfunction. The diverse pathophysiology of primary DCM and RCM suggests distinct underlying genetic mechanisms. A well-established disease gene for DCM and RCM is cardiac troponin I3 (*TNNI3*), which causes dominant and recessively inherited forms. In children, bi-allelic truncating *TNNI3* variants have typically been associated with DCM, and heterozygous missense *TNNI3* variants are associated with RCM. We report a 2-year-old female with severe RCM that is genetically caused by a homozygous *TNNI3* nonsense variant, c.406C>T (p.Arg136∗), which results in a more distal (C-terminal) truncation than most previously reported disease-associated nonsense variants. In myocardial biopsies of the patient, TNNI3 protein abundance was diminished, suggesting that residual TNNI3 function may underlie RCM, while TNNI3 absence causes DCM. The RCM in this patient was treatment refractory and resulted in a heart transplant at the age of 28 months. Overall, recessive TNNI3 protein truncation causes severe pediatric RCM, suggesting that the allelic status, type of genetic alteration, and length of TNNI3 protein truncation determine cardiomyopathy onset and subtype manifestation.

## Teaser

Cardiac troponin I3 (TNNI3) determines heart muscle contractility by regulating Ca^2+^ sensitivity of the sarcomere. Homozygous loss of TNNI3 protein function typically induces pediatric dilated cardiomyopathy (DCM) by decreasing Ca^2+^ sensitivity. This study demonstrates that homozygous TNNI3 truncation also induces early-onset pediatric restrictive cardiomyopathy (RCM), suggesting increased Ca^2+^ sensitivity.

## Introduction

Cardiomyopathy is a heterogeneous group of myocardial disorders that cause structural and functional abnormalities of the heart muscle. Different types of cardiomyopathies are distinguished by heart morphology and pathophysiology and include hypertrophic (HCM), dilated (DCM), arrhythmogenic (ACM), and restrictive (RCM) cardiomyopathy. RCM is rare in children and the least frequent cardiomyopathy in adults.^1^^,^^2^ In pediatric patients, RCM arises most frequently in the first 2 years of life and accounts for a low percentage of all individuals affected by cardiomyopathy, depending on the specific cohort.^2^^,^^3^ Clinically, RCM is characterized by diastolic dysfunction due to impaired muscle relaxation and myocardial stiffness, resulting in restrictive ventricular filling.^4^^,^^5^ Of note, pediatric RCM is frequently characterized by progressive heart failure and therapy resistance.^2^^,^^3^^,^^6^^,^^7^ RCM may arise in familial forms, spontaneously due to *de novo* variants, or as part of genetic syndromes such as Fabry and Danon disease or transthyretin amyloidosis.^8^ The most frequent RCM disease genes are *FLNC*, *MYH7*, *TNNI3*, *TNNT2*, and *TPM1*.^2^^,^^7^^,^^9^ Among pediatric patients with cardiomyopathy, heterozygous missense variants in *TNNI3* are estimated to account for >30% of individuals with RCM.^2^^,^^7^

The TNNI3 protein constitutes a component of the 3-part troponin complex, which also includes cardiac troponin T2 (TNNT2) and cardiac troponin C1 (TNNC1).^10^ The troponin complex associates with actin and tropomyosin of the sarcomere thin filament and regulates Ca^2+^ sensitivity during striated muscle contraction.^10^ Cardiac troponin I proteins isoforms (adult TNNI3 and fetal TNNI1) control sarcomere contraction by regulating actin-myosin cross-bridging in response to increased intracellular Ca^2+^ levels.^10^ *TNNI3* comprises eight exons, and most disease-causing missense variants accumulate in the C-terminal exons 7 and 8.^11^^,^^12^ The TNNI3 C terminus mediates TNNC1 and actin-tropomyosin protein interaction, together facilitating myosin-actin binding in response to Ca^2+^ stimuli.^13^

TNNI3 serves as inhibitory subunit of the troponin complex determining in response to Ca^2+^ actin-myosin interaction and contractility. Consequently, genetic variation in TNNI3 may decrease or increase Ca^2+^ sensitivity of the sarcomere.^11^^,^^12^ Decreased TNNI3-associated sarcomere Ca^2+^ sensitivity diminishes contractility, resulting in systolic dysfunction and DCM.^12^ Severely increased TNNI3-associated Ca^2+^ sensitivity diminishes sarcomere relaxation, resulting in stiff heart muscle, diastolic dysfunction, and RCM.^11^^,^^12^ Moderate increase of TNNI3 Ca^2+^ sensitivity results in HCM, which is the typical consequence of dominant *TNNI3* missense variants.^11^^,^^12^ Homozygous *TNNI3* variants that abolish TNNI3 protein expression (N-terminal protein-truncating variants) cause DCM due to reduced sarcomere Ca^2+^ sensitivity, diminished myosin-actin binding, and poor contractility.^14^ Of note, among published affected individual with homozygous truncating TNNI3 variants, all patients developed DCM but not RCM (Table 1). Here, we show that a homozygous TNNI3 truncating variant induces early-onset severe RCM.

**Table 1:** Summary of affected individuals with homozygous, compound heterozygous *TNNI3* variants

Case	Phenotype	Sex	Age initial diagnosis	Outcome	TNNI3 variant protein	*TNNI3* variant transcript	TNNI3 exon	Zygosity	ClinVar ID pathogenicity	Parents
#1	DCM	M F	27 years 29 years	HTX no	p.Ala2Val	c.5C>T^a^	exon 1	hom	VUS^b^	no HP
#2	DCM	F	1 year	deceased	p.Ala8Ala splice effect/TNNI3_ex1-8del^c^	c.24G>A/TNNI3_ex1-8del^c^	exon 2	comp. het^c^	no	N/D
#3	LVNC	F	12 months	deceased	splice effect	c.24+2T>A	intron 2	hom	P, VUS^b^	N/D
#4	DCM	F	12 months	N/D	splice effect	c.24+2T>A	intron 2	hom	P, VUS^b^	N/D
#5	DCM, myocarditis	–	3 years, 2 years	HTX, HTX	p.Lys50Lys, splice effect	c.150G>A	exon 4	hom	VUS	no HP
#6	DCM, myocarditis	F	3 years	deceased	p.Lys50Lys, splice effect	c.150G>A	exon 4	hom	VUS	N/D
#7	DCM	F	3 years	HTX	p.Arg69Alafs∗8	c.204del	exon 5	hom	P, LP, VUS^b^	N/D
#8	DCM	F	2 months	HTX	p.Arg69Alafs∗8	c.204del	exon 5	hom	P, LP, VUS^b^	mother no HP, father HP
#9	DCM	M M	6 months 7 months	deceased deceased	p.Arg69Alafs∗8	c.204del	exon 5	hom	P, LP, VUS^b^	no HP
#10	DCM	F	11 months	deceased	p.Arg69Alafs∗8	c.204del	exon 5	hom	P, LP, VUS^b^	N/D
#11	DCM	M	14 months	HTX	p.Arg69Alafs∗8	c.204del	exon 5	hom	P, LP, VUS^b^	N/D
#12	DCM	F	9 months	N/D	p.Arg69Alafs∗8	c.204del	exon 5	hom	P, LP, VUS^b^	N/D
#13	DCM	F	10 months	N/D	p.Arg69Alafs∗8	c.204del	exon 5	hom	P, LP, VUS^b^	N/D
#14	DCM	M	6 months	HTX	p.Arg69Alafs∗8	c.204del	exon 5	hom	P, LP, VUS^b^	N/D
#15	DCM	F F F	12 months 13 months 13 months	deceased deceased deceased	p.Arg69Alafs∗8	c.204del	exon 5	hom	P, VUS^b^	no HP
#16	HCM	M	38 years	no	p.Arg79Cys	c.235C>T	exon 5	hom	B, LB, VUS^b^	N/D
#17	HCM	F M	N/D N/D	N/D	p.Arg79Cys/p.Ala157Val	c.235C>T/c.470C>T	exon 5/7	comp. het	B, LB, VUS^b^ P	N/D
#18	DCM/LVNC	M	6 months	ND	p.Leu88Trpfs∗27	c.258del	exon 5	hom	P, VUS^b^	no HP
#19	DCM (myocarditis)	F	7 months	HTX	p.Arg98^a^	c.292C>T	exon 6	hom	P, VUS^b^	N/D
#20	RCM	F	24 months	HTX, LTX, deceased	p.Arg136^a^	c.406C>T	exon 7	hom	VUS	N/D
#21	HCM	N/D	N/D	N/D	p.Arg141Gln	N/D	exon 7	hom	LP, P^b^	N/D
#22	HCM HCM	F M	17 years 15 years	– ICD	p.Arg162Trp	N/D	exon 7	hom	LP, P^b^	no HP
#23	HCM	F	17 years	ICD	p.Arg162Trp	N/D	exon 7	hom	LP, P^b^	no HP
#24	DCM	M	1 month	deceased	p.Glu182Lys	c.544G>A	exon 7	hom	LP, P^b^	N/D
#25	HCM RCM RCM	M F^d^ F^d^	42 years 41 years 45 years	no	p.Asp196His	c.586G>C	exon 8	hom	VUS	no HP
#26	DCM	F	14 month	deceased at 19 months	–	11 kb deletion at 19q13.42 comprising *TNNT1* exons 1–9, *TNNI3* exon 8	exon 8	hom	no	N/D

A version of this table including relevant reference citations is provided as Table S1. M, male; F, female; hom, homozygous; comp. het, compound heterozygous; HTX, heart transplantation; LTX, liver transplantation; LVNC, left ventricular non-compaction cardiomyopathy; ICD, implantable cardioverter defibrillator; N/D, not determined; HP, heart phenotype; B, benign; LB, likely benign; VUS, variant of unknown significance; LP, likely pathogenic; P, pathogenic.

a This variant was in the original publication,^15^ described as c.4C>T. The triplet at this position is GCG, coding for alanine. We corrected this typo according to the published amino acid exchange p.Ala2Val.

b Conflicting interpretations in ClinVar.

c The variant p.Ala8Ala occurs compound heterozygously with a deletion of TNNI3 exons 1–8. The variant interrupts the canonical donor splice site of *TNNI3* intron 2, inducing premature stop of translation.

d Individuals are dizygotic twin sisters.

## Material and methods

### Clinical case report

The proband was consented to a research protocol allowing for the release of tissue for study and publication of this report (Cincinnati Children's Hospital Medical Center, Cincinnati, USA). Clinical exome sequencing of the proband and her mother was performed by GeneDx (Gaithersburg, Maryland, USA). The proband’s clinical chart was reviewed and relevant information extracted for summary in this report.

### Heart tissue analysis

Heart biopsies were sampled from the proband’s explanted heart and subjected to paraformaldehyde fixation and paraffin embedding. Paraffin sections were cut with a 5 μm thickness and processed according to standard protocols. Three independent heart biopsies from pediatric patients without myocardial disease served as controls. For immunofluorescence analysis, tissue sections were probed with anti-TNNI3 (Thermo Fisher Scientific, PA5-28964), anti-DES (Dako, M0760) primary antibodies, and anti-rabbit Alexa Fluor 568 and anti-mouse Alexa Fluor 647 secondary antibodies. The primary, polyclonal anti-TNNI3 antibody detects epitopes within full-length human TNNI3 (1–210 aa). Nuclei and plasma membranes were stained with 4′,6-diamidin-2-phenylindol (DAPI) Alexa Fluor 405 and wheat germ agglutinin (WGA) Alexa Fluor 488, respectively. Imaging of immunofluorescence staining was performed with a four-channel laser-scanning microscope (LSM700, Zeiss, Germany) under identical imaging conditions. For quantitative analysis, the image intensity was measured with ZEN 3.0 (Zeiss, Germany). Analysis of heart tissue with transmission electron microscopy (TEM) was done according to standard protocols in the Division of Pathology, Cincinnati Children’s Hospital Medical Center (https://www.cincinnatichildrens.org/research/divisions/p/pathology).

### Literature review for homozygous TNNI3 variants

Clinical affected individual with cardiomyopathy due to homozygous TNNI3 variant were identified from a literature research (Pubmed database: https://pubmed.ncbi.nlm.nih.gov/, Clarivate Web of Science) and ClinVar (ClinVar database: https://www.ncbi.nlm.nih.gov/clinvar/). Each case description was validated in depth, and core data were assembled in Table 1. These data include cardiomyopathy type, sex, age of initial diagnosis, clinical outcome, genetic *TNNI3* variant information, ClinVar pathogenicity, and whether the variant carriers developed a heart phenotype.

## Results

### Clinical case presentation

The female proband presented for emergency care at 26 months of age with sudden-onset left-sided facial and extremity weakness in the setting of a prior known history of alpha-1-antitrypsin deficiency (AATD). Head CT confirmed an acute ischemic stroke. A chest X-ray revealed an enlarged cardiac silhouette with interstitial opacities. Echocardiogram identified severe RCM with severely dilated right and left atria (103 mL/m^2^), no left ventricular (LV) hypertrophy (interventricular septum z = −2.3, LV posterior wall z = −0.7), LV dilation (diastolic dimension z = 1.0), and normal systolic function (LV ejection fraction: 57%). She had moderate tricuspid regurgitation and severe mitral valve regurgitation. In addition, an unusual morphology of the chordae tendineae of both atrioventricular valves was noted, leading to a tethering phenomenon. Cardiac catheterization revealed elevated right ventricular and LV end diastolic pressures (15 and 29 mmHg by wedge pressure), elevated mean pulmonary artery pressure (35 mmHg), and low-normal cardiac index (2.65 L/min/m^2^). Given her hemodynamics and the prognosis of RCM, she was listed for heart transplantation (HTX). Clinical genetics consultation was requested after the diagnosis of RCM. The proband’s birth history was notable for vacuum-assisted vaginal delivery at 39 weeks. Her newborn hearing and newborn metabolic screens were normal. She was small for gestational age (2.5 kg, Z = −1.8). She was admitted to the neonatal intensive care unit for elevated liver enzymes and cholestasis and ultimately diagnosed with AATD based on low alpha-1-antitrypsin levels (23 mg/dL, normal: 90–200 mg/dL) and confirmatory genetic testing (*SERPINA1*, *AAT*, c.1096G>A [GenBank: NM_000295.4] [p.Glu366Lys]). Development was delayed, with onset of walking between 13 and 16 months and use of only 5–10 single words at 24 months. Physical examination at 26 months revealed microcephaly (44.5 cm head circumference, Z = −2.0), a broad nose, low-set and posteriorly rotated ears that are simple and dysplastic, and thin, fine hair. Physical features were not suggestive of a specific genetic syndrome. The maternal family history was negative for any form of cardiomyopathy or sudden death. Paternal family history was unknown. Due to the diagnosis of RCM, patient 1-III:1 was listed for HTX (3 weeks after RCM diagnosis) and received a transplant at 2 years 4 months of age, ∼2 months after RCM diagnosis. At 53 months of age, patient 1-III:1 had a liver transplant (LTX) due to progressive liver disease related to her AATD. She experienced multiple complications and passed away several days after transplant; her cause of death was determined to be necrotizing pneumonia with Gram-negative sepsis and disseminated intravascular coagulopathy.

### Genetic analysis

**Figure 1:**
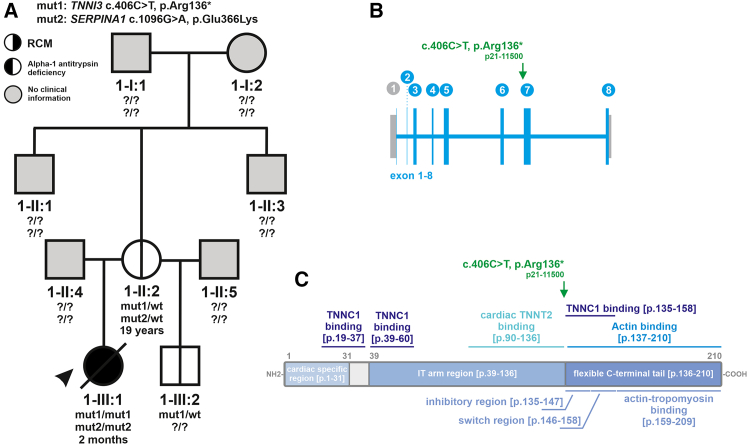
Genetic analysis (A) Pedigree of family 1, including index patient 1-III:1 (black arrow). The filling of the symbols indicates clinical status for restrictive cardiomyopathy (RCM; black right half) and alpha-1 antitrypsin deficiency (black left half). Gray filling indicates the absence of clinical information. The genotypes are shown as mut1 (*TNNI3* c.406C>T [p.Arg136∗]) and mut2 (*SERPINA1* c.1096G>A [p.Glu366Lys]). (B) Scheme of the human *TNNI3* gene depicting all exons and the genetic variant detected in patient 1-III:1. (C) Scheme of the human TNNI3 protein highlights protein regions with their functional implication and association with other sarcomere proteins. The TNNI3 p.Arg136∗ variant abolishes the entire flexible C-terminal tail, including the actin-tropomyosin binding regions.

Given the severity of her RCM in conjunction with abnormal growth parameters and developmental delay, clinical exome sequencing on the mother and patient was ordered (Figure 1A). Exome sequencing identified a homozygous *TNNI3* variant, c.406C>T (GenBank: NM_000363.4) (p.Arg136∗), that localizes in exon 7 and creates a premature translational stop signal (Figures 1B and 1C). The truncated TNNI3 p.Arg136∗ protein lacks the entire C terminus that mediates the actin and actin-tropomyosin interaction. The performing genetics laboratory classified the genetic result as a variant of uncertain significance (VUS). Segregation analysis revealed that the mother (1-II:2) and maternal half-brother (1-III:2) were both heterozygous for the TNNI3 p.Arg136∗ variant (Figure 1A). The mother (1-II:2, age 20) and half-brother (1-III:2, age 21 months) demonstrated normal heart function on echocardiography.

Evaluation of the homozygous *TNNI3* p.Arg136∗ variant using the ClinGen gene-specific American College of Medical Genetics (ACMG) criteria for TNNI3 gained the terms PM2_supp, PM3, and PM4 (ClinGen database: https://cspec.genome.network/cspec/ui/svi/doc/GN098).

### TNNI3 protein analysis

**Figure 2:**
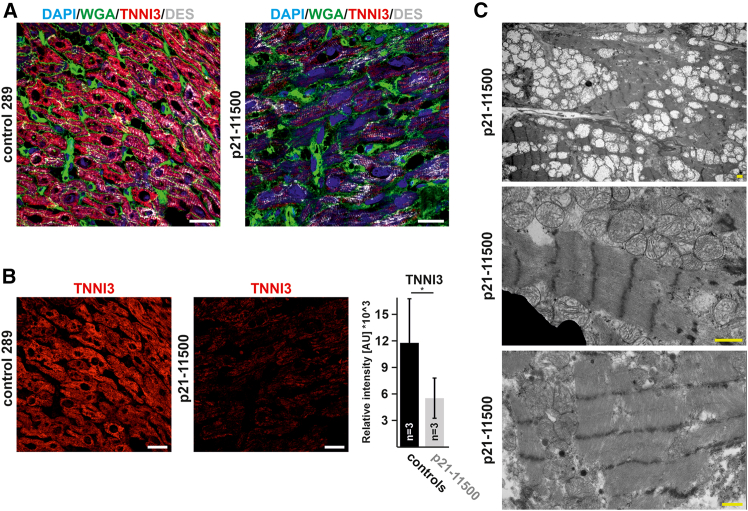
Functional analysis of heart tissue (A) Immunostaining was performed on heart tissue from patient 1-III:1 (p21-11500) and control subjects. Diminished TNNI3 immunostaining was detected in p21-11500 tissue. Staining occurred for nuclei with 4′,6-diamidin-2-phenylindol (DAPI; blue), plasma membranes with wheat germ agglutinin (WGA; green), cardiac troponin I3 (TNNI3; red), and desmin (DES; gray). Imaging was performed with 4-laser confocal microscopy. Scale bar: 20 μm. (B) Quantitative analysis of heart tissue sections measuring the abundance of the TNNI3 protein (red). Statistical analysis was performed with an unpaired *t* test; *p* < 0.05. (C) Transmission electron microscopy (TEM) was performed with immersion-fixed heart samples from the explanted heart. TEM demonstrated myofibrillar disarray with abnormal sarcomeres and mitochondrial hyperplasia. Higher power resolution revealed abnormal sarcomeres with indistinct M-lines and irregular Z-bands. Scale bars: 1 μm.

To clarify the effect of the bi-allelic truncating *TNNI3* variant in the patient, we quantified expression of the mutant TNNI3 p.Arg136∗ protein. Three independent heart tissue samples from patient 1-III:1 (p21-11500, *n* = 3) were compared with age-matched control subjects without myocardial disease (*n* = 3). Immunostaining of the TNNI3 protein and subsequent confocal imaging under identical exposure conditions revealed lower staining intensity in p21-11500 patient biopsies than in control subjects (Figure 2A). Quantitative image analysis measured approximately 50% reduction of TNNI3 staining intensity. Furthermore, cardiomyocytes appeared less regularly organized. Of note, immunostaining still detects TNNI3 protein in the heart tissue samples of the p21-11500 patient. This suggests that the homozygous truncated TNNI3 protein is synthesized by cardiomyocytes and is, to a certain degree, stable (Figure 2B). Pathological TEM from samples of the explanted heart showed indistinct and poorly formed M lines, irregular Z bands, and multifocal contraction artifacts (Figure 2C). Mitochondrial hyperplasia was noted. Based on the diminished protein level, the ACMG terms for the variant TNNI3 p.Arg136∗ could be expanded to PS3, PM2_supp, PM3, and PM4, resulting in a final evaluation of likely pathogenic (LP), class 4.

## Discussion

### Homozygous variants in TNNI3 are a determinant of severe, early-onset pediatric cardiomyopathy

Our study adds to the recent literature reporting homozygous variants in *TNNI3* as a cause of severe, early-onset pediatric cardiomyopathy.^3^^,^^16^ We provide additional evidence that certain recessive *TNNI3* variants can cause severe pediatric RCM. The association of bi-allelic *TNNI3* variants with RCM was previously documented in a family carrying the *TNNI3* missense variant p.Asp196His.^17^ Only recently was another affected individual with early-onset pediatric RCM, due to the same homozygous truncating variant TNNI3 p.Arg136∗ as in our affected individual, published.^18^ This association is further strengthened by several reports detecting heterozygous *TNNI3* missense variants in patients with RCM.^3^^,^^19^^,^^20^^,^^21^ Most individuals with HCM demonstrated adult disease onset due to homozygous *TNNI3* missense variants, suggesting that altered TNNI3 function, but not loss of function, induces HCM. In contrast, early pediatric (<2 years of age) DCM develops most frequently due to bi-allelic TNNI3 protein truncation (suspected loss of function). This reveals a striking difference in age-dependent phenotype development in response to homozygous truncating or missense *TNNI3* variants. Of note, TNNI3 protein-truncating variants causing DCM induce severe pediatric courses frequently resulting in HTX or premature death (Table 1).^2^^,^^3^^,^^7^^,^^22^

Postnatal absence of TNNI3 is mechanistically critical, as it physiologically replaces the fetal TNNI isoform TNNI1. TNNI3 is required for cardiomyocyte maturation during postnatal cardiac development and in stem cell-derived cardiomyocytes.^23^ Through cardiomyocyte development, TNNI switching adapts Ca^2+^ sensitivity, resistance to hypoxia/acidosis, and cardiac responsiveness to adrenergic stimulation.^24^ Recently, we showed that loss of TNNI3 is compensated with elevated TNNI1 levels in pediatric cardiac tissue, illustrating that defective molecular adaptation of cardiomyocytes to the postnatal environment impairs proper postnatal heart function.^3^ Recent studies analyzing the TNNI3 variant p.Arg170Trp using induced pluripotent stem cell-derived cardiomyocytes (iPSC-CMs) modeled diastolic dysfunction as a key parameter of RCM and rescued the impaired relaxation phenotype genetically.^25^^,^^26^ Overall, there are key molecular differences between adult and pediatric TNNI3-associated disease that result in cardiomyopathies of differing severities.^14^^,^^16^ Pediatric RCM and DCM due to homozygous TNNI3 truncating variants are severe entities that require careful clinical handling.

### Does TNNI3 haploinsufficiency cause cardiomyopathy?

A growing body of genetic studies highlights the relevance of homozygous, bi-allelic *TNNI3* alleles for the development of cardiomyopathy, specifically in pediatric patients (Table 1).^3^^,^^15^^,^^27^^,^^28^^,^^29^ However, most frequently, *TNNI3*-associated HCM is due to heterozygous missense variants and follows an autosomal-dominant trait.^30^ ClinGen states little evidence for haploinsufficiency in *TNNI3* (ClinGen database: https://search.clinicalgenome.org/kb/genes/HGNC:11947). This aligns with our review, which identified that none of the clinically evaluated *TNNI3* carrier parents of pediatric patients with recessive *TNNI3* disease had developed cardiomyopathy (Table 1). One limitation of our current understanding of parental *TNNI3* variant carriers is the infrequent reporting of outcomes and clinical assessment. A recent study reviewing dominant and recessive cardiomyopathies also reported that TNNI3 protein-truncating variants did not induce cardiomyopathy in the heterozygous state.^14^ Overall, this suggests that protein-truncating *TNNI3* variants cause cardiomyopathy only in the homozygous state.^14^

One limitation of our study is that we present only one patient with RCM due to the homozygous TNNI3 p.Arg136∗ variant. Interestingly, another recent report identified a girl with early-onset RCM who was homozygous for the TNNI3 p.Arg136∗ variant.^18^ Another limitation is that we could not monitor the cardiac *TNNI1*-to-*TNNI3* isoform switch in fresh frozen tissue by measuring transcript levels.

In conclusion, this study highlights that the homozygous TNNI3 p.Arg136∗ variant is a rare cause of severe pediatric RCM. We speculate that the TNNI3 p.Arg136∗ protein increases sarcomere Ca^2+^ sensitivity, leading to stiff muscle, diastolic dysfunction, and finally RCM. Further work is needed to delineate the diverse development of cardiomyopathy subtypes and disease onset due to *TNNI3* truncating or missense variants. The diversity in cardiomyopathy subtype expression and onset suggests differential mechanistic implications, possibly due to diminished or altered TNNI3 function.

## Data and code availability

Primary data are available upon reasonable request to the corresponding authors (K.N.W. and J.K.).

## Acknowledgments

We thank the family for participating in this study. We thank the Advanced Light Microscopy Technology Platform of the Max-Delbrück-Center for Molecular Medicine, Berlin, for the general and technical support (Anca Margineanu and Anje Sporbert). We thank Olaf Grisk for critically reading the manuscript. Funding was provided by the 10.13039/100010447German Centre for Cardiovascular Research, and Deutsches Zentrum für Herz-Kreislauf-Forschung e.V. (10.13039/100010447DZHK), partner site Berlin, supported S.K. with research grants 81Z0100216, 81X2100230, 81Z0100301, and 81Z3100333. Clinical research enrollment was supported in part through the Cincinnati Children’s Heart Institute Research Core.

## Declaration of interests

The authors declare no competing interests.

## Web resources

PubMed, https://pubmed.ncbi.nlm.nih.gov/

ClinVar, https://www.ncbi.nlm.nih.gov/clinvar/

## Supplemental information

Document S1. Table S1 and supplemental notes

Document S2. Article plus supplemental information
