# Supplementary material for: A homozygous variant in cardiac troponin I3, TNNI3, causes severe pediatric restrictive cardiomyopathy
Source: HGG Adv. 2026 Mar 30;7(3):100598. doi: 10.1016/j.xhgg.2026.100598 (PMC13096904; doi:10.1016/j.xhgg.2026.100598)
Supplement: Document S1. Table S1 and supplemental notes [file mmc1.pdf]

HGGA, Volume 7

Supplemental information

A homozygous variant in cardiac troponin I3, TNNI3, causes severe pediatric restrictive cardiomyopathy

Jirko Kühnisch, Cara L. Barnett, Josephine Brendel, Lara Berklite, Chet Villa, Wenke Seifert, Sabine Klaassen, Karin Klingel, and K. Nicole Weaver

Table 1: Summary of cases with homozygous, compound heterozygous *TNNI3* variants (full version including References)

Case	Phenotype	Sex	Age initial diagnosis	Outcome	TNNI3 variant protein	*TNNI3* variant transcript	TNNI3 exon	Zygosity	ClinVar ID pathogenicity	Parents	Reference
**#1**	DCM	M<br>F	27 years<br>29 years	HTX<br>no	p.Ala2Val	c.5C>T*	Exon 1	hom	VUS[#]	no HP	1
**#2**	DCM	F	1 year	deceased	p.Ala8Ala splice effect/<br>TNNI3_ex1-8del[$]	c.24G>A/<br>TNNI3_ex1-8del[$]	Exon 2	comp. het[$]	no	n.d.	2
**#3**	LVNC	F	12 months	deceased	splice effect	c.24+2T>A	Intron 2	hom	P, VUS[#]	n.d.	3
**#4**	DCM	F	12 months	n.d.	splice effect	c.24+2T>A	Intron 2	hom	P, VUS[#]	n.d.	4
**#5**	DCM,<br>myocarditis	-	3 years,<br>2 years	HTX,<br>HTX	p.Lys50Lys,<br>splice effect	c.150G>A	Exon 4	hom	VUS	no HP	5
**#6**	DCM,<br>myocarditis	F	3 years	deceased	p.Lys50Lys,<br>splice effect	c.150G>A	Exon 4	hom	VUS	n.d.	6
**#7**	DCM	F	3 years	HTX	p.Arg69Alafs*8	c.204del	Exon 5	hom	P, LP, VUS[#]	n.d.	5
**#8**	DCM	F	2 months	HTX	p.Arg69Alafs*8	c.204del	Exon 5	hom	P, LP, VUS[#]	mother no HP<br>father HP	5
**#9**	DCM	M<br>M	6 months<br>7 months	deceased<br>deceased	p.Arg69Alafs*8	c.204del	Exon 5	hom	P, LP, VUS[#]	no HP	5
**#10**	DCM	F	11 months	deceased	p.Arg69Alafs*8	c.204del	Exon 5	hom	P, LP, VUS[#]	n.d.	5
**#11**	DCM	M	14 months	HTX	p.Arg69Alafs*8	c.204del	Exon 5	hom	P, LP, VUS[#]	n.d.	3,7
**#12**	DCM	F	9 months	n.d.	p.Arg69Alafs*8	c.204del	Exon 5	hom	P, LP, VUS[#]	n.d.	4
**#13**	DCM	F	10 months	n.d.	p.Arg69Alafs*8	c.204del	Exon 5	hom	P, LP, VUS[#]	n.d.	4
**#14**	DCM	M	6 months	HTX	p.Arg69Alafs*8	c.204del	Exon 5	hom	P, LP, VUS[#]	n.d.	8
**#15**	DCM	F<br>F<br>F	12 months<br>13 months<br>13 months	deceased<br>deceased<br>deceased	p.Arg69Alafs*8	c.204del	Exon 5	hom	P, VUS[#]	no HP	9
**#16**	HCM	M	38 years	no	p.Arg79Cys	c.235C>T	Exon 5	hom	B, LB, VUS[#]	n.d.	10
**#17**	HCM	F<br>M	n.d.<br>n.d.	n.d.	p.Arg79Cys/<br>p.Ala157Val	c.235C>T/<br>c.470C>T	Exon 5/7	comp. het	B, LB, VUS[#]<br>P	n.d.	11
**#18**	DCM/LVNC	M	6 months	n.d.	p.Leu88Trpfs*27	c.258del	Exon 5	hom	P, VUS[#]	no HP	12
**#19**	DCM<br>(myocarditis)	F	7 months	HTX	p.Arg98*	c.292C>T	Exon 6	hom	P, VUS[#]	n.d.	8
**#20**	RCM	F	24 months	HTX, LTX<br>deceased	p.Arg136*	c.406C>T	Exon 7	hom	VUS	n.d.	this study

**#21**	HCM	n.d.	n.d.	n.d.	p.Arg141Gln	n.d.	Exon 7	hom	LP, P[#]	n.d.	[13]
**#22**	HCM<br>HCM	F<br>M	17 years<br>15 years	-<br>ICD	p.Arg162Trp	n.d.	Exon 7	hom	LP, P[#]	no HP	[14]
**#23**	HCM	F	17 years	ICD	p.Arg162Trp	n.d.	Exon 7	hom	LP, P[#]	no HP	[15]
**#24**	DCM	M	1 month	deceased	p.Glu182Lys	c.544G>A	Exon 7	hom	LP, P[#]	n.d.	[16]
**#25**	HCM<br>RCM<br>RCM	M<br>F[§]<br>F[§]	42 years<br>41 years<br>45 years	no	p.Asp196His	c.586G>C	Exon 8	hom	VUS	no HP	[17]
**#26**	DCM	F	14 month	deceased with 19 months	-	11 kb deletion at 19q13.42 comprising *TNNT1* exons 1–9, *TNNI3* exon 8	Exon 8	hom	no	n.d.	[18]

* This variant was in the original publication [1] described as c.4C>T. The triplet at this position is GCG coding for alanine. We corrected this typo according to the published amino acid exchange p.Ala2Val. [§] Individuals are dizygotic twin sister. [$] The variant p.Ala8Ala occurs compound heterozygous with a deletion of TNNI3 exon 1-8. The variant interrupts the canonical donor splice site of *TNNI3* intron 2 inducing premature stop of translation. [#] Conflicting interpretations in ClinVar. HTX - heart transplantation. LTX - liver transplantation. ICD - implantable cardioverter defibrillator. n.d. - not determined. HP - heart phenotype. B - benign. LB - likely benign, VUS - variant of unknown significance, LP - likely pathogenic, P - pathogenic.

Additional clinical details

The homozygous SERPINA1 p.Glu366Lys variant induces alpha-1 antitrypsin deficiency

The patient 1-III:1 was diagnosed with AATD terminally resulting in LTX. Genetic analysis identified the variant SERPINA1/AAT p.E366K in patient 1-III:1 homozygously. SERPINA1 is synthesized in the liver and serves as serine proteases inhibitor (SerPIn) inactivating for instance elastase, plasmin, or thrombin. SERPINA1 protects tissues from uncontrolled damage due to serine proteases, e.g. neutrophil elastase. Only recently, a functional study systematically assessed the biochemistry of AATD associated SERPINA1 variants.[19] The variant SERPINA1 p.E366K (or Z-allele) lacks neutrophil elastase inhibitory activity, polymerizes/aggregates in the hepatocyte endoplasmic reticulum, and shows low monomer abundance after hepatocyte secretion.[19] These biochemical properties make the SERPINA1 p.E366K highly pathogenic. Clinically, this variant is associated with chronic obstructive pulmonary disease (COPD) and liver cirrhosis. Thus, the homozygous state of the SERPINA1 p.E366K variant is highly pathogenic and explains liver disease in patient 1-III:1.[20] Of note, the available enzyme replacement therapy is not effective in patients with the SERPINA1 p.E366K variant due to pathological intracellular aggregation in hepatocytes.[21] Development of personalized therapies will help to handle such severe cases of AATD in the future.[19]

References:

1. Murphy, R.T., Mogensen, J., Shaw, A., Kubo, T., Hughes, S., and McKenna, W.J. (2004). Novel mutation in cardiac troponin I in recessive idiopathic dilated cardiomyopathy. Lancet *363*, 371-372. 10.1016/S0140-6736(04)15468-8.
2. Yu, T., Yan, F., Xu, Y., Hunag, Y., Gong, H., Zhao, P., Sun, D., Zhang, Y., Zhang, F., and He, X. (2023). Identification of a novel TNNI3 synonymous variant causing intron retention in autosomal recessive dilated cardiomyopathy. Gene *856*, 147102. 10.1016/j.gene.2022.147102.
3. Kuhnisch, J., Herbst, C., Al-Wakeel-Marquard, N., Dartsch, J., Holtgrewe, M., Baban, A., Mearini, G., Hardt, J., Kolokotronis, K., Gerull, B., et al. (2019). Targeted panel sequencing in pediatric primary cardiomyopathy supports a critical role of TNNI3. Clinical genetics *96*, 549-559. 10.1111/cge.13645.
4. Pezzoli, L., Pezzani, L., Bonanomi, E., Marrone, C., Scatigno, A., Cereda, A., Bedeschi, M.F., Selicorni, A., Gasperini, S., Bini, P., et al. (2021). Not Only Diagnostic Yield: Whole-Exome Sequencing in Infantile Cardiomyopathies Impacts on Clinical and Family Management. J Cardiovasc Dev Dis *9*. 10.3390/jcdd9010002.
5. Janin, A., Perouse de Montclos, T., Nguyen, K., Consolino, E., Nadeau, G., Rey, G., Bouchot, O., Blanchet, P., Sabbagh, Q., Cazeneuve, C., et al. (2022). Molecular Diagnosis of Primary Cardiomyopathy in 231 Unrelated Pediatric Cases by Panel-Based Next-Generation Sequencing: A Major Focus on Five Carriers of Biallelic TNNI3 Pathogenic Variants. Mol Diagn Ther *26*, 551-560. 10.1007/s40291-022-00604-3.
6. Belkaya, S., Kontorovich, A.R., Byun, M., Mulero-Navarro, S., Bajolle, F., Cobat, A., Josowitz, R., Itan, Y., Quint, R., Lorenzo, L., et al. (2017). Autosomal Recessive Cardiomyopathy Presenting as Acute Myocarditis. Journal of the American College of Cardiology *69*, 1653-1665. 10.1016/j.jacc.2017.01.043.
7. Seidel, F., Holtgrewe, M., Al-Wakeel-Marquard, N., Opgen-Rhein, B., Dartsch, J., Herbst, C., Beule, D., Pickardt, T., Klingel, K., Messroghli, D., et al. (2021). Pathogenic Variants Associated With Dilated Cardiomyopathy Predict Outcome in Pediatric Myocarditis. Circ Genom Precis Med *14*, e003250. 10.1161/CIRCGEN.120.003250.
8. Sorrentino, U., Gabbiato, I., Canciani, C., Calosci, D., Rigon, C., Zuccarello, D., and Cassina, M. (2023). Homozygous TNNI3 Mutations and Severe Early Onset Dilated Cardiomyopathy: Patient Report and Review of the Literature. Genes (Basel) *14*. 10.3390/genes14030748.
9. Kraoua, L., Louati, A., Ahmed, S.B., Abida, N., Khemiri, M., Menif, K., Mrad, R., Zaffran, S., and Jaouadi, H. (2024). Homozygous TNNI3 frameshift variant in a consanguineous family with lethal infantile dilated cardiomyopathy. Mol Genet Genomic Med *12*, e2486. 10.1002/mgg3.2486.
10. Zhang, L., Ding, F., Ren, Z., Cheng, W., Dai, H., Liang, Q., Kong, F., Xu, W., Wang, M., Zhang, Y., and Tao, Q. (2025). Mechanisms of pathogenicity in the hypertrophic cardiomyopathy-associated TNNI3 c.235C > T variant. International journal of cardiology *419*, 132627. 10.1016/j.ijcard.2024.132627.
11. Zheng, H., Huang, H., Ji, Z., Yang, Q., Yu, Q., Shen, F., Liu, C., and Xiong, F. (2016). A Double Heterozygous Mutation of TNNI3 Causes Hypertrophic

Cardiomyopathy in a Han Chinese Family. Cardiology *133*, 91-96. 10.1159/000440877.

12. Mehaney, D.A., Haghighi, A., Embaby, A.K., Zeyada, R.A., Darwish, R.K., Elfeel, N.S., Abouelhoda, M., El-Saiedi, S.A., Gohar, N.A., and Seliem, Z.S. (2022). Molecular analysis of dilated and left ventricular noncompaction cardiomyopathies in Egyptian children. Cardiology in the young *32*, 295-300. 10.1017/S1047951121002055.
13. Mogensen, J., Hey, T., and Lambrecht, S. (2015). A Systematic Review of Phenotypic Features Associated With Cardiac Troponin I Mutations in Hereditary Cardiomyopathies. The Canadian journal of cardiology *31*, 1377-1385. 10.1016/j.cjca.2015.06.015.
14. Gray, B., Yeates, L., Medi, C., Ingles, J., and Semsarian, C. (2013). Homozygous mutation in the cardiac troponin I gene: clinical heterogeneity in hypertrophic cardiomyopathy. International journal of cardiology *168*, 1530-1531. 10.1016/j.ijcard.2012.12.008.
15. Maron, B.J., Maron, M.S., and Semsarian, C. (2012). Double or compound sarcomere mutations in hypertrophic cardiomyopathy: a potential link to sudden death in the absence of conventional risk factors. Heart rhythm : the official journal of the Heart Rhythm Society *9*, 57-63. 10.1016/j.hrthm.2011.08.009.
16. Li, X., Dai, L., and Zhang, J. (2023). Case Report: Mutation in TNNI3(c. 544G>A): a novel likely pathogenic mechanism of neonatal dilated cardiomyopathy. Front Pediatr *11*, 1291609. 10.3389/fped.2023.1291609.
17. Pantou, M.P., Gourzi, P., Gkouziouta, A., Armenis, I., Kaklamanis, L., Zygouri, C., Constantoulakis, P., Adamopoulos, S., and Degiannis, D. (2019). A case report of recessive restrictive cardiomyopathy caused by a novel mutation in cardiac troponin I (TNNI3). BMC medical genetics *20*, 61. 10.1186/s12881-019-0793-z.
18. Streff, H., Bi, W., Colon, A.G., Adesina, A.M., Miyake, C.Y., and Lalani, S.R. (2019). Amish nemaline myopathy and dilated cardiomyopathy caused by a homozygous contiguous gene deletion of TNNT1 and TNNI3 in a Mennonite child. European journal of medical genetics *62*, 103567. 10.1016/j.ejmg.2018.11.001.
19. Zhao, P., Wang, C., Sun, S., Wang, X., and Balch, W.E. (2024). Tracing genetic diversity captures the molecular basis of misfolding disease. Nature communications *15*, 3333. 10.1038/s41467-024-47520-0.
20. Ruiz, M., Lacaille, F., Schrader, C., Pons, M., Socha, P., Krag, A., Sturm, E., Bouchecareilh, M., and Strnad, P. (2023). Pediatric and Adult Liver Disease in Alpha-1 Antitrypsin Deficiency. Semin Liver Dis *43*, 258-266. 10.1055/a-2122-7674.
21. Strnad, P., McElvaney, N.G., and Lomas, D.A. (2020). Alpha(1)-Antitrypsin Deficiency. The New England journal of medicine *382*, 1443-1455. 10.1056/NEJMra1910234.
